# Maternal outcomes in subsequent delivery after previous obstetric anal sphincter injury (OASI): a multi-centre retrospective cohort study

**DOI:** 10.1007/s00192-019-03983-0

**Published:** 2019-06-22

**Authors:** Joanna Caroline D’Souza, Ash Monga, Douglas G. Tincello, Abdul H. Sultan, Ranee Thakar, Timothy C. Hillard, Stephanie Grigsby, Ayisha Kibria, Clare F. Jordan, Christopher Ashmore

**Affiliations:** 1 Faculty of Medicine, University of Southampton, University Hospitals NHS Foundation Trust, Southampton, UK; 2 Princess Anne Hospital, University Hospitals NHS Foundation Trust, Southampton, UK; 3 Department of Health Sciences, College of Life Sciences, University of Leicester, Leicester, UK; 4 University Hospitals of Leicester NHS Trust, Leicester, UK; 5 Croydon Health Services NHS Trust, Croydon, UK; 6 Poole Hospital NHS Foundation Trust, Poole, UK

**Keywords:** Obstetric anal sphincter injuries, Perineal trauma, Mediolateral episiotomy, Recurrent obstetric anal sphincter injury

## Abstract

**Introduction and hypothesis:**

Women with a history of obstetric anal sphincter injury (OASI) are at increased risk of recurrence (rOASI) at subsequent delivery; however, evidence regarding the factors influencing this risk is limited. Furthermore, little is known about what factors influence the decision to alternatively deliver by elective caesarean section (ELLSCS).

**Methods:**

Retrospective univariate and multivariate logistic regression analysis of prospectively collected data from four NHS electronic maternity databases including primiparous women sustaining OASIS during a singleton, term, cephalic, vaginal delivery between 2004 and 2015, who had a subsequent delivery.

**Results:**

Two thousand two hundred seventy-two women met the criteria; 10.2% delivering vaginally had a repeat OASI and 59.4% had a second-degree tear. Women having an ELLSCS were more likely to be Caucasian, older, have previously had an operative vaginal delivery (OVD) and have a more severe degree of OASI. Positive predictors for rOASI were increased birth weight and maternal age at both index and subsequent deliveries, a more severe degree of initial OASI and Asian ethnicity. The overall mediolateral episiotomy (MLE) rate was 15.6%; 77.2% of those who had an episiotomy sustained no spontaneous perineal trauma. Only 4.4% of women with a rOASI had an MLE, whilst the MLE rate was 16.9% in those without a recurrence (*p* < 0.001). MLE decreased the risk of rOASI by 80%. Birth weight > 4 kg increased the risk 2.5 fold.

**Conclusions:**

Women with previous OASIS are at an increased risk of recurrence. A more liberal use of MLE during subsequent vaginal delivery could significantly reduce the risk of recurrence.

## Introduction

More than 85% of women sustain some form of perineal trauma during vaginal childbirth in the UK, which equates to approximately 350,000 injuries a year [1, 2]. Perineal trauma can occur spontaneously or is iatrogenic, when an episiotomy is performed. Obstetric anal sphincter injuries (OASIS), the most severe form of spontaneous perineal trauma, can result in significant morbidity and are a contributing factor to long-term anal incontinence [3]. Rates of OASIS in the UK have steadily increased from 1.8% in 2000 to 5.9% in 2012, probably because of better detection and reporting rather than poor quality care or a change in risk factors [4]. The overall median OASIS incidence is 2.9% (range 0–8%), with primipara at 3.6-fold higher risk than multipara (6.1% vs. 1.7%, *p* < 0.01) [5].

A recent systematic review and meta-analysis revealed a recurrent OASI (rOASI) rate of 6.8% [6]. Operative vaginal delivery (OVD) (forceps greater than vacuum extraction), previous 4th-degree tear, birth weight of successive infant > 4 kg, Asian ethnicity and shoulder dystocia are associated with an increased risk of rOASI [6, 7].

The use of prophylactic episiotomy in prevention of rOASI is not clear [8]. The aforementioned meta-analysis showed no association; however, there was significant heterogeneity of the data collection as all episiotomy techniques were included. One recent UK-based cohort study has shown a potential protective effect of mediolateral episiotomy (MLE) against rOASI (aOR 0.66; 95% CI 0.58–0.75) [7].

The current recommendation is to counsel women with a history of OASI about the risk of recurrence (5–7%) and potential for de novo or worsening of faecal symptoms (incidence of 17%) [8, 9]. Elective lower segment caesarean section (ELLSCS) should be considered in symptomatic women or in those with sphincter defects on endoanal ultrasound and/or abnormally low anal manometry pressures [8]. However, a more recent prospective follow-up study evaluating the impact of mode of subsequent delivery on anorectal symptoms and physiology concluded that symptomatic women with normal anorectal physiology could subsequently deliver vaginally and still achieve good anorectal outcomes [10].

The primary outcome measure of this study was to investigate the grade of perineal trauma at subsequent delivery after an obstetric anal sphincter injury (OASI) and explore what maternal, intrapartum and neonatal factors influence the risk of rOASI, namely the use of MLE. Our secondary outcome measure was to explore what factors influence the likelihood of subsequently delivering by ELLSCS.

## Methods

This is a retrospective population-based cohort study which analysed prospectively collected data from maternity databases and paper records from the following National Health Service (NHS) Trusts in the UK; University of Southampton NHS Foundation Trust, Croydon Health Services NHS Trust, Poole Hospital NHS Foundation Trust and University Hospitals of Leicester NHS Trust.

At each site the Information Management and Technology (IM&T) Specialist for Maternity sampled from the Trust’s electronic maternity database all women whom had sustained an OASI between January 2004 and December 2015. These were then filtered to only include OASI sustained in primparous women who had a singleton, term, cephalic delivery. Members of the clinical research team at each Trust then prospectively selected those with a recorded subsequent delivery. Information regarding the initial and subsequent delivery was extracted manually from electronic and paper hospital records. Data sets from all four Trusts were then collated and analysed at the host site (University of Southampton NHS Foundation Trust). All degrees of perineal trauma involving the anal sphincter muscles were combined into one variable in the analysis.

Analysis was carried out using IBM SPSS v.24. Univariate analysis was carried out to compare maternal, neonatal and intrapartum factors at initial and subsequent delivery between women sustaining rOASI at subsequent delivery with those who did not. The Kolmogorov-Smirnov test was used to determine the distribution of continuous data; parametric data were analysed using independent samples *t*-test and non-parametric data, the Mann-Whitney U test. Chi-square test was used to analyse categorical data. Multivariate analysis was carried out through binary logistic regression to calculate the adjusted independent odds ratio (OR) of OASIS at subsequent delivery, including factors reaching statistical significance (*p* < 0.05). Further univariate analysis explored the subsequent deliveries in women who had sustained OASIS at first delivery, comparing the maternal, neonatal and intrapartum factors of those with a further vaginal delivery with those having an ELLSCS.

The study was granted full ethical approval by the NHS Health Research Authority, reference no. 16/SC/0126. Only anonymised data were used, so informed consent was not required.

## Results

**Fig. 1:**
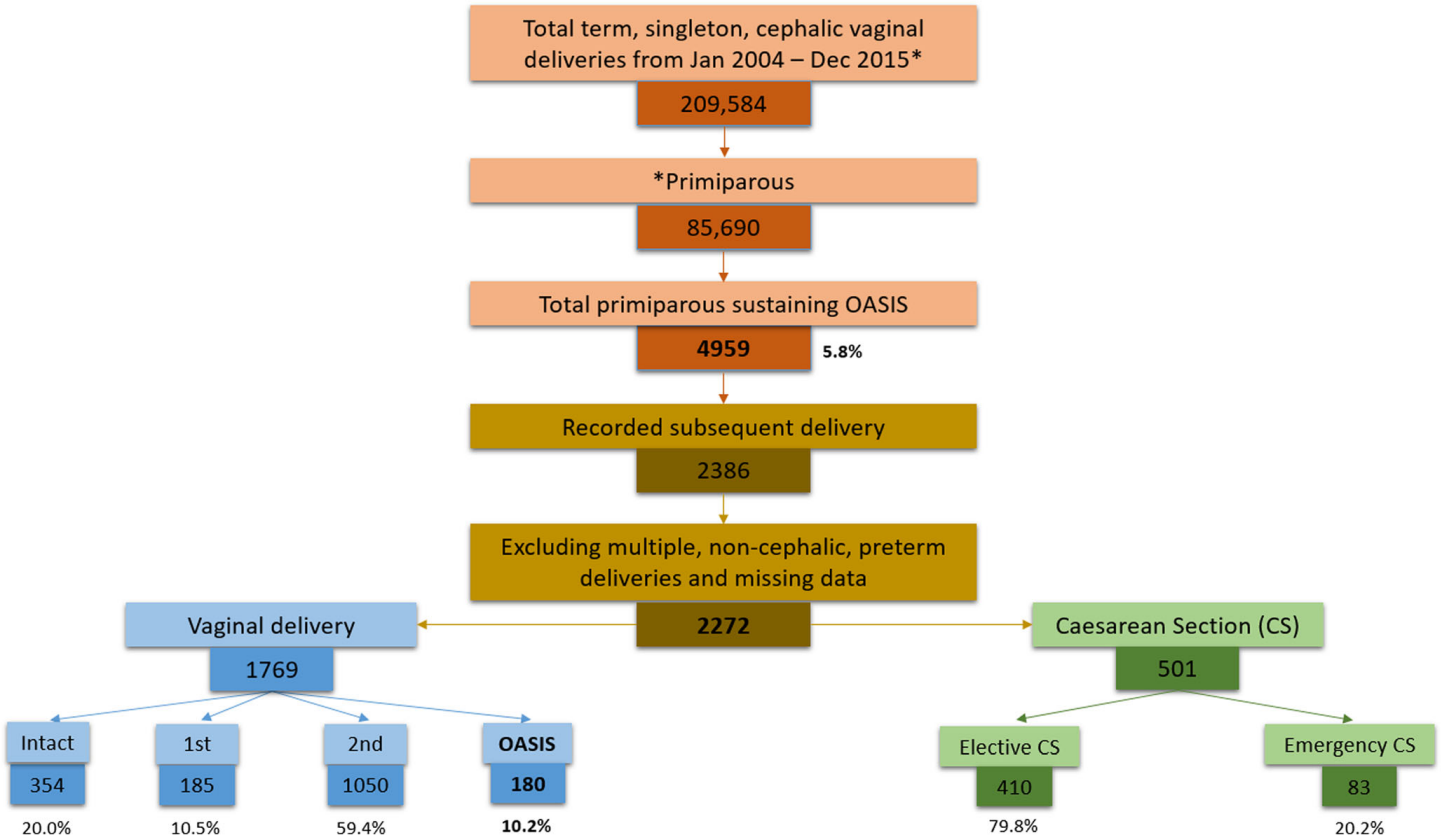
Schematic diagram representing birthing outcomes and perineal condition at repeat vaginal birth

In the 12-year time period, there were 209,584 singleton, term, cephalic vaginal deliveries of which 40.9% were primiparous women. The overall prevalence of OASIS was 3.1%; 77.3% of all OASIS were sustained by primiparous women at a rate of 5.8%, which is significantly greater than both the multiparous and overall rates of OASIS, 1.2% (difference 4.6%, 95% CI 4.5, 4.8) and 3.1% (difference 2.7%, 95% CI 2.6, 2.9), respectively. Of the primiparous women sustaining OASIS 48.1% had a further recorded delivery. Having excluded all multiple, preterm and non-cephalic deliveries, and incomplete records, the study population was 2272. Of these, 77.9% (*n* = 1769) had a subsequent vaginal delivery, of which 95.3% were by normal vaginal delivery (NVD), 2.5% had vacuum extraction and 2.1% were delivered by forceps. The OASI recurrence rate was 10.2%. The most common perineal injury after previous OASIS was a second-degree perineal trauma (59.4% of births). See Fig. 1 and Table 1 for overall delivery and perineal outcomes.

**Table 1:** Perineal condition and incidence of episiotomy at subsequent vaginal delivery

Perineal Condition	Count	Percentage that had episiotomy	
No spontaneous trauma	354 (20.0%)	213 (60.2%)	268 (16.9%)
1^st^	185 (10.5%)	9 (4.9%)	
2^nd^	1050 (59.4%)	46 (4.4%)	
OASIS	180 (10.2%)	8 (4.4%)	
Total	**1769**		

Univariate analyses are shown in Table 2, comparing maternal, neonatal and intrapartum factors concerning the risk, or not, of sustaining repeat OASIS at subsequent vaginal delivery. Those sustaining rOASI were significantly older at both the index and subsequent delivery (28 vs. 27 years, *p* = 0.013 and 31 vs. 30, *p* = 0.010) and had significantly heavier babies at subsequent delivery (3625 vs. 3502.5, *p* = 0.001), with a greater proportion > 4 kg (25.0% vs. 14.3%, *p* < 0.001). Women with rOASI were more likely to have had a more severe degree of anal sphincter injury at their first delivery. No difference was seen when analysing the mode of delivery or whether the subsequent delivery was post-term.

**Table 2:** Comparison of women sustaining a rOASI at subsequent delivery with those that did not

		Repeat OASIS at subsequent VD	No OASIS at subsequent VD	p-value
		(n=180)	(n=1589)	
Ethnicity (n=1591: 165 rOASI, 1589 no rOASI)	Caucasian Asian Black	105 (63.9%) 53 (32.1%) 7 (4.2%)	987 (69.2%) 360 (25.9%) 70 (4.9%)	p=0.225^a^
Age	**Index delivery** Median (years) **Subsequent delivery** Median (years)	28 (15 – 40) 31 (18 – 41)	27 (15 – 48) 30 (17 – 50)	**p=0.013**^b^ **p=0.010**^b^
Birth weight (g)	**Index delivery** Mean (g) % over 4Kg **Subsequent delivery** Median (g) % over 4Kg	3459.5 (±468.01) 19 (10.6%) 3625 (2512 – 5440) 45 (25.0%)	3420.8 (±455.61) 174 (11.0%) 3502.5 (2030 – 6480) 228 (14.3%)	p=0.296^c^ p=0.872^a^ p=0.001^b^ **p<0.001**^a^
Degree of OASIS at 1^st^ delivery (n=902: 104 rOASI, 798 no rOASI)	Overall comparison **3a** – < 50% of EAS involved **3b** – ≥ 50% of EAS involved **3c** – EAS and IAS involvement **4**^**th**^ – 3c + anorectal mucosa	37 (35.6%) 50 (48.1%) 10 (9.6%) 7 (6.1%)	428 (53.6%) 268 (33.6%) 53 (6.6%) 49 (6.1%)	**p=0.006**^a^
Operative VD (As a percentage of all deliveries)	**Index delivery** **Subsequent delivery**	51 (28.3%) 6 (3.3%)	464 (29.2%) 77 (4.8%)	p=0.808^a^ p=0.363^a^
Gestation	Post-term (>40 weeks)	99 (55.0%)	762 (48.0%)	p=0.073^a^
Episiotomy	Overall rate NVD Forceps delivery Vacuum extraction	8 (4.4%) 6 (3.4%) 1 (50.0%) 1 (25.0%)	268 (16.9%) 202 (13.4%) 34 (94.4%) 32 (78.0%)	**p<0.001**^a^ **p<0.001**^a^ **p=0.023**^a^ **p=0.022**^a^

^a^Chi-square, ^b^ Mann-Whitney U Test (non-parametric data), ^c^ Independent t-test (parametric)

The overall MLE rate at subsequent delivery was 15.6% (276/1769) and was carried out in 81.9% of OVDs (92.1% forceps, 73.3% vacuum extraction) and 12.3% (208/1687) of NVDs. Four out of 15 (26.7%) women having an operative vaginal delivery (OVD) without an episiotomy had a repeat sphincter injury compared with 2.9% (2/68) of those with an MLE. MLE was protective against OASIS (*p* < 0.001; difference 12.4%, 95% CI 6.8, 18.0). This was regardless of delivery mode [NVD *p* < 0.001 (13.4% of repeat OASIS without MLE vs. 3.4% of repeat OASIS with MLE, 95% CI 4.5, 15.0), forceps *p* = 0.02 (difference 30.5%, 95% CI 4.1, 56.8), vacuum extraction p = 0.02 (difference 22.0%, 95% CI 3.2, 40.8)]; 77.2% of those with MLE sustained no spontaneous perineal trauma. Only 4.4% of women with rOASI had an MLE; 2.9% (8/276) of those with MLE had a rOASI. Table 1 details the incidence of episiotomy with each degree of perineal trauma sustained. The number of MLEs required to prevent one OASI is eight [NNT = 1/ARR = 1/(0.169–0.044)] when including all modes of VD, ten if the delivery was an NVD [NNT = 1/(0.134–0.034)].

The factors which remained independently associated with the risk of OASIS after binary logistic regression are shown in Table 3. These included the age of the mother at subsequent delivery, proportion of babies weighing > 4 kg at subsequent delivery, degree or severity of OASIS at initial delivery and whether an episiotomy was performed at subsequent delivery. The analysis of odds ratios revealed that episiotomy at subsequent delivery decreased the risk of repeat OASIS by 80%, whereas birth weight > 4 kg increased the risk of repeat OASI by 2.5 fold.

**Table 3:** Factors independently associated with the risk of rOASI after binary logistic regression

	Odds ratio	p-value	95% CI
Age of mother at subsequent delivery (years)	1.05	0.032	1.004 – 1.097
If birthweight >4Kg at subsequent delivery (%)	2.51	<0.001	1.534 – 4.122
Degree of OASIS at initial delivery (%)	1.57	0.001	1.240 -1.989
Episiotomy at subsequent delivery (%)	0.21	<0.001	0.080 – 0.524

The caesarean section (LSCS) rate at subsequent birth was 22.1%, of which 79.8% were elective. The analyses in Table 4 compare the women having a further VD with those having an ELLSCS. Those having an emergency LSCS were excluded from the analysis as the indication for LSCS was unknown.

**Table 4:** Comparison of women with subsequent VD vs. ELLSCS

		VD at subsequent delivery	ELLSCS at subsequent delivery	p-value
Ethnicity (n=1963: 1598 VD, 365 ElLSCS)	Caucasian Asian Black	1098 (68.7%) 423 (26.5%) 77 (4.8%)	313 (85.8%) 48 (13.2%) 4 (1.1%)	**p<0.001**^a^
Age	**Index delivery** Median **Subsequent delivery** Median	28 (15 – 48) 30 (17 – 50)	29 (15 – 42) 32 (16 – 46)	**p<0.001**^b^ **P<0.001**^b^
Birth weight (g)	**Index delivery** Mean % over 4Kg **Subsequent delivery** Median % over 4Kg	3450.2 (±454.14) 193 (10.9%) 3520 (2030 - 6480) 273 (15.4%)	3577.5 (±455.61) 73 (17.7%) 3480 (2000 – 4820) 47 (11.4%)	**p<0.001**^c^ **p<0.001**^a^ **p=0.001**^b^ **p=0.042**^a^
Mode of delivery at 1^st^ delivery	Operative VD	517 (29.1%)	176 (42.7%)	**p<0.001**^a^
Degree of OASIS at 1^st^ delivery (n=1112: 910 VD, 202 ElLSCS)	Overall comparison **3a** **3b** **3c** **4**^**th**^	468 (51.4%) 318 (34.9%) 65 (7.1%) 59 (6.5%)	54 (26.7%) 88 (43.6%) 21 (10.4%) 39 (19.3%)	**p<0.001**^a^

^a^Chi-square, ^b^ Mann-Whitney U Test (non-parametric data), ^c^ Independent t-test (parametric)

Significant variation was seen when comparing the mode of subsequent delivery across the categories of ethnicity. Caucasian women were 2.2 times and 4.5 times more likely to have had an ELLSCS than Asian and Black women, respectively (22.2% vs. 10.2% and 4.9% as proportion of women from each ethnic category). Women having an ELLSCS were significantly older at both initial and subsequent delivery. They also had heavier babies at first delivery (3577 vs. 3450, *p* < 0.001), with a significantly greater proportion weighing > 4 kg (17.7% vs. 10.9%, *p* < 0.001). Women having ELLSCS had a worse grade of OASIS at initial delivery and were 1.5 times more likely to have had an operative vaginal delivery than those having repeat vaginal delivery.

## Discussion

### Main findings

This study aimed to assess whether there are any factors which influence the risk of women sustaining repeat OASIS.

Our primary finding was that women with a history of previous OASI had a greater risk of rOASI than both primiparous and other multiparous women without previous OASI. The rate of OASIS in these populations was similar to previously quoted rates, but we found the recurrence rate to be far greater than those previously quoted [6, 7, 11]. Recurrence was more likely with increased maternal age, if the subsequent infant had a birth weight > 4 kg and a more severe degree of OASI at index delivery. MLE was shown to be protective against rOASI regardless of the delivery mode. Those having an ELLSCS were more likely to be older at both index and subsequent delivery than those having a further VD, to be Caucasian, to have had an OVD at index delivery and to have sustained a more severe degree of OASI.

### Strength and limitations

This study’s strength lies in the fact that data collection was achieved through manual, prospective examination of electronic- and paper-based birthing records of 2272 women having sustained an OASI over a 12-year period. Data collection in this manner removes potential inaccuracies associated with incomplete or incorrect electronic coding, which has been highlighted as a limitation of previous large database studies [5, 7, 12]. However, one potential limitation of our study was that the process of identification of those meeting the inclusion criteria was electronic and hence at risk of being subject to incorrect coding. Data were also extracted from four different Trust-based maternity databases. We believe that we have largely overcome any potential coding inaccuracies by manual prospective collection of data concerning the subsequent delivery and retrospective review of the electronically extracted data of the index delivery. Approximately 1% of collated data were incomplete and excluded from analysis, and an entire year’s data were excluded from one site because of errors in coding associated with a changeover of the maternity database that year.

A further limitation was that individual cases were subject to bias in clinical decision making as the data encompass the practice of many different clinicians at four individual sites over a 12-year time frame. However, we believe it safe to assume practitioners were working in accordance with nationally recognized guidelines, hence validating the merging of the data sets. Unfortunately, we do not know whether the angle at which the MLEs were performed was at the recommended 60^o^ as the patients were not examined. However, given the fact that it has been established that an episiotomy cut at a 60^o^ angle is protective, the impact of ensuring a 60^o^ angle can only enhance its beneficial effect. We are also aware that the extent to which OASI preventative measures, such as manual perineal protection, are used may vary between the different sites. Additionally, the indication for ELLSCS was unknown. Therefore, the analysis was based on the assumption that the reason for ELLSCS over VD was the resultant effect of the OASI sustained at index delivery, e.g., symptoms of sphincter injury or abnormal anorectal physiology. It would have been of interest to also compare the rates of induction of labour, length of second stage, birthing position and foetal position, but this was not within the scope of this study.

### Interpretation

This research provides new information about the mode of delivery at subsequent birth after previous OASI and the proportion of the other less severe forms of perineal trauma (e.g. those with no spontaneous trauma, 1st-degree or 2nd-degree perineal trauma). When comparing women who sustained rOASI with those who did not, a greater incidence of repeat trauma was seen amongst those of Asian ethnicity. This is in line with other studies that have shown ethnic variation in perineal length, pelvic anatomy and tissue composition and resultant differences in predisposition to birth trauma [7, 13–16].

In agreement with earlier studies exploring the risk of OASIS in the primiparous population, we found both macrosomia and increased maternal age carried through to the subsequent delivery as positive predictors of rOASI [11, 12, 17]. We also found that women with a more severe degree of OASIS at initial delivery were at increased risk of a rOASI. In contrast to previous literature, no association was seen between mode of delivery at initial and subsequent vaginal delivery and risk of a recurrence [11].

MLE has been shown to be protective against sphincter damage at OVD and a recent review quotes an overall 40–50% reduction in risk of OASI with MLE [16, 18]. The use of MLE in the prevention of rOASI was less clear [6]. Although the episiotomy rate at subsequent delivery in this study was lower than the national rate of 20.2% [5], the cohort not sustaining rOASI were significantly more likely to have had an MLE regardless of delivery mode. Overall, eight episiotomies would need to be performed to prevent one OASI (inclusive of all delivery modes), ten if the delivery was non-operative. Unfortunately numbers were too low to reliably comment on the NNT at OVD. A recent large national cohort study and the current national guidelines regarding the prevention of primary OASI promote the use of MLE at OVD [4, 8]. This study goes one step further in suggesting MLE also has a preventative effect against rOASI, regardless of the mode of VD. The use of MLE after previous OASI returns the rate of OASI at subsequent delivery to the overall UK national rate of 2.9% (1.7% for multiparous women) [5]. Furthermore, it is of interest to note that in addition to being protective against rOASI, MLE is also protective against all lesser degrees of perineal trauma.

Multivariate logistic regression has become the analytic tool of choice in retrospective studies and was useful in this study to determine the factors independently associated with the risk of OASIS [19]. Most strikingly, MLE was associated with an 80% reduction in the risk of rOASI. This is the first published study to make this conclusion and could go some way in providing the required evidence to update the current recommendations in favour of the use of prophylactic MLE in the prevention of rOASI [8]. Although it is important to recognize that MLE is not without potential complications such as long-term symptoms of perineal pain and dyspareunia, we would agree with previous research that the morbidity has less of an impact than an OASI, and we would expect this to be even more so the case in the event of a recurrence [18].

An interesting observation was seen when analysing the delivery mode after OASI across the ethnic categories; a greater proportion of Caucasian women had a subsequent ELLSCS than both Asian and Black. A possible interpretation is that women of ethnic minority groups are more likely to underreport symptoms, opt for a more natural approach and be less inclined to accept recommendations—observations seen in other areas of clinical medicine [20]. There is also the possibility of institutional bias against ethnic groups due to inadequacies in the clinicians’ abilities to effectively facilitate discussions with those of different cultures. Without information regarding the indication for ELLSCS, this observation is entirely speculative.

Those having ELLSCS at subsequent birth were significantly older at both index and subsequent delivery, which correlates with the observed impact of maternal age on obstetric outcome and the increased likelihood of requiring a CS [21]. This also supports previous research regarding age-related change in perineal collagen composition, which could predispose both the initial injury, resultant symptoms and the recommendation for a subsequent ELLSCS [22]. Due to the gestation at which the ELLSCSs would have taken place, these women had significantly lighter babies than those having a further VD.

The results of this study support the notion based on linearity regarding the degree of sphincter involvement and severity of symptoms and hence worse damage resulting in the recommendation for a subsequent ELLSCS. These women had significantly heavier babies at first delivery, with a greater proportion weighing > 4 kg, and they were 1.5 times more likely to have had an operative vaginal delivery, factors associated with greater severity of trauma [7, 17].

## Conclusion

Women with previous OASIS are at an increased risk of recurrence, further predisposing them to anal sphincter dysfunction. Increased maternal age and birth weight and severity of tear at index delivery are positive predictors for rOASI. More liberal use of mediolateral episiotomy could decrease the risk of recurrence by 80%. This information will be useful in aiding clinical decision-making and counselling of women who decide to have a further vaginal delivery after an OASI.
